# Recontact and follow-up for individuals with germline pathogenic variants in hereditary breast and ovarian cancer susceptibility genes: a UK Cancer Genetics Group consensus meeting

**DOI:** 10.1136/jmg-2025-110979

**Published:** 2025-10-01

**Authors:** Joseph Christopher, Katharine Edgerley, Beth McIldowie, Rosalyn Jewell, Zoe Kemp, Katie Snape, Helen Hanson, Consensus meeting attendees, Julian Barwell

**Affiliations:** 1 East Anglian Medical Genetics Service, Cambridge University Hospitals NHS Foundation Trust, Cambridge, UK; 2 Department of Genomic Medicine, National Institute for Health Research Cambridge Biomedical Research Centre, University of Cambridge, Cambridge, UK; 3 St George’s University of London, London, England, UK; 4 All Wales Medical Genomics Service, Cardiff, UK; 5 Leeds Clinical Genomics Service, Leeds, UK; 6 Cancer Genetics Unit, Royal Marsden Hospital Sutton, Sutton, UK; 7 South West Thames Regional Genomics Service, London, UK; 8 Peninsula Regional Clinical Genetics Service, Royal Devon University Healthcare NHS Foundation Trust, Exeter, UK; 9 Department of Clinical and Biomedical Sciences, University of Exeter Medical School, Exeter, UK

**Keywords:** Genetic Predisposition to Disease, Genetic Testing, Genetic Counseling, Neoplasms, Patient Care

## Abstract

**Background:**

Routine genetic testing for germline pathogenic variants (GPVs) in cancer susceptibility genes (CSGs) in individuals with suspected hereditary cancer risk, and subsequent cascade testing in close relatives, has led to a significantly increased number of individuals identified to be at increased lifetime cancer risk in the UK. These individuals have differing clinical needs over their lifetime to implement cancer surveillance and discuss risk-reducing interventions, to advise on risk to close relatives and counsel on reproductive options. Regional clinical genetics services across the UK have implemented a range of clinical pathways to manage this patient cohort, with differences in practice across centres. Our aim was to provide consensus guidelines on best practice for recontact and follow-up for individuals with GPVs in *BRCA1*, *BRCA2*, *PALB2*, *ATM*, *CHEK2*, *BARD1*, *BRIP1*, *RAD51C, RAD51D* and mismatch repair (MMR) genes (*MLH1*, *MSH2*, *MSH6* and *PMS2*) to ensure appropriate lifelong clinical management.

**Methods:**

The UK Cancer Genetics Group convened a virtual consensus meeting involving key stakeholders. Consensus for best practice guidance was achieved through premeeting surveys, structured discussions and in-meeting polling.

**Results:**

We identified variability in the extent of active recontact and follow-up for individuals with a GPV in CSGs across the UK. The availability of resources was a key determinant of the level of follow-up currently provided. We achieved consensus on best practice guidelines on key time points for recontact for those with a GPV in a CSG, processes for referral to specialist services and follow-up, the preferred method for recontact and the content of information given during recontact.

**Conclusion:**

The consensus meeting broadly supported recontact and follow-up for most individuals with a GPV in a CSG. The consensus achieved by the multidisciplinary and expert group of healthcare professionals gives clear guidance on the long-term management of this patient cohort at increased lifetime risk of cancer and highlights the additional resources that would be required to effectively deliver this.

**KEY MESSAGE**

- Increasing numbers of individuals with germline pathogenic variants (GPVs) in cancer susceptibility genes (CSGs) are being identified in clinical practice.
- These individuals have differing clinical needs over their lifetime with respect to cancer surveillance, discussion of risk-reducing interventions and advice on risk to family members or reproductive options.
- In the UK, there have been no national recommendations for recontact or follow up of individuals once they have received their results and discussed gene-specific cancer risks and clinical management.
- The UK Cancer Genetics Group convened a virtual consensus meeting with key stakeholders to provide consensus guidelines on best practice for recontact and follow-up for individuals with GPVs in *BRCA1, BRCA2, PALB2, ATM, CHEK2, BARD1, BRIP1, RAD51C, RAD51D* and mismatch repair (MMR) genes (*MLH1, MSH2, MSH6* and *PMS2*) to ensure appropriate lifelong clinical management.
- Consensus for best practice was reached, including key time points for recontact and follow up for those with a GPV in a CSG, processes for referral to specialist services and follow-up, the preferred method for recontact, and the content of information given during recontact. It was acknowledged that additional resource will be required to effectively deliver this in practice.

## Introduction

 Cancer is unfortunately common, with one in two people in the UK diagnosed with cancer within their lifetime. While the majority of cancer occurs as a result of environmental, hormonal and lifestyle factors, approximately 5%–10% of all cancers are caused by inherited genetic alterations (germline pathogenic or likely pathogenic variants (GPV)) in cancer susceptibility genes (CSG), also commonly referred to as ‘cancer predisposition genes’. At present, there are approximately 130 such genes and associated cancer predisposition syndromes described. When GPVs are present in CSGs, an individual will have an increased lifetime risk of developing cancer, with their overall cancer risk dependent on their genotype (table 1) as well as several modifying factors, including family history of cancer, hormone status, lifestyle and environmental factors. Regional Clinical Genetics services (RCGS) specialise in the evaluation, estimation and management of heritable cancer risk. An expanding repertoire of early detection and prevention management options is available, which includes enhanced cancer surveillance programmes, risk-reducing surgery and therapies and patient education/symptom awareness. Furthermore, there are implications for existing family members and reproductive options.^1 2^ The personal burden and unmet information needs for individuals with a GPV in a CSG are often high and are often exacerbated by a lack of support through overstretched healthcare resources.^3^,^5^ The relevance and implementation of these interventions vary across an individual’s lifetime, necessitating ongoing follow-up and comprehensive counselling services. National guidelines curated by the UK Cancer Genetics Group (UKCGG) exist for the recommended clinical management of individuals with a GPV in a CSG^6^ (online supplemental table 1).

**Table 1:** Summary of lifetime penetrance estimates for individuals with a heterozygous germline pathogenic variant in the cancer susceptibility genes listed

Cancer susceptibility gene	Breast cancer risk to age 80 years	Ovarian cancer risk to age 80 years	Endometrial cancer risk to age 75 years	Other associated cancer risk
Population risk	Approximately 10%	Approximately 1.5%	Approximately 2%	
*BRCA1*	72% (65%–79%)	44% (36%–53%)		Male breast and pancreatic
*BRCA2*	69% (61%–77%)	17% (11%–25%)		Male breast, prostate and pancreatic
*PALB2*	53% (44%–63%)	5% (2%–10%)		Male breast and pancreatic
*CHEK2*	26% (22%–29%)*			Prostate
*ATM*	24% (19%–28%)*			Pancreatic and prostate
*BARD1*	24% (14%–32%)*			
*RAD51C*	21% (15%–29%)*	11% (6%–21%)*		
*RAD51D*	20% (14%–28%)*	13% (7%–23%)*		
*BRIP1*		5%–12%		
*MLH1*		8% (5%–12%)†	37% (31%–44%)	Colorectal, gastrointestinal, urothelial, prostate, pancreatic, etc
*MSH2*		13% (9%–20%)†	44% (36%–53%)	Colorectal, gastrointestinal, urothelial, prostate, pancreatic, etc
*MSH6*		6% (3%–15%)†	46% (36%–57%)	Colorectal, gastrointestinal, urothelial, pancreatic, etc
*PMS2*		Near population†	21% (9%–47%)	Colorectal

Population cancer risk estimates from personal communication with CRUK Cancer Intelligence team (November 2022). Cancer risk estimates for *BRCA1*/*BRCA2* based on prospective *BRCA1* and *BRCA2* consortium data;^21^ *PALB2* based on segregation analysis,^22^ *CHEK2*, *ATM* and *BARD1* breast cancer risk based on Breast Cancer Association Consortium case-control analysis;^23^ *BRIP1* risk estimates are based on the data summarised by Domchek *et al*^24^; *RAD51C* and *RAD51D* based on segregation analysis;^25^ and mismatch repair genes based on the Prospective Lynch Syndrome Database.^26^

* Risk estimates can be significantly altered by factors such as family history, modifiable risk factors and polygenic risk score.

† Risk estimates to age 75 years.

Testing for monogenic causes of hereditary cancer susceptibility has expanded significantly in recent years due to the broadening of eligibility criteria for germline testing in England^7^ and the identification of germline variants through tumour sequencing^8^ and population-based pilots such as the National Health Service (NHS) Jewish BRCA programme.^9^ The increase in diagnosis of individuals with hereditary cancer susceptibility is set to continue, with the range of risk management options likely to increase in number and complexity, and personalised cancer risk estimates likely to vary by context (clinical ascertainment vs population testing). The long-term management of these individuals will be coordinated by RCGS with collaboration across other specialist services. The establishment of the National Inherited Cancer Predisposition Register (NICPR) in England, in collaboration with NHS England and the National Disease Registration Service, will create a central register of all individuals in England with a monogenic cause of inherited cancer predisposition and provide a resource that could help facilitate recontact and follow-up.^10^

To date, RCGS have implemented a variety of measures to manage their local cohorts of at-risk individuals, but long-term routine follow-up is not typically offered following discussion of results, cancer risk, symptom awareness and appropriate onward referral for surveillance or risk-reducing interventions. This is complicated somewhat by the wide range of ages and circumstances through which an individual is identified to have a GPV in a CSG. Examples include an incidental finding during genetic testing for non-cancer indications, including prenatal testing, diagnostic testing (eg, a 30-year-old with breast cancer) or cascade testing for a known familial variant in a relative (eg, parent, grandparent or sibling). The content of discussion at the point of diagnosis requires significantly different approaches in different situations, as well as considerations for future recontact and follow-up.

To recognise and address these differences and form consensus best practice guidelines for recontact and follow-up of at-risk individuals, the UKCGG, in collaboration with the Cancer Research UK-funded CanGene-CanVar programme, organised a national consensus meeting, held virtually, with the following aims:

1. Gain consensus for a national approach to recontact individuals with a monogenic inherited cancer predisposition.
2. Define a minimum standard for coordination of care and ongoing follow-up through RCGS.

Specialists from RCGS across all four nations, along with representatives from patient groups and NHS England, were invited to participate in the meeting. The consensus discussion focused on specific genes comprising the breast and ovarian cancer susceptibility genes listed on the National Genomic Test Directory inherited ovarian cancer (R207 V.4.0) and inherited breast cancer and ovarian cancer (R208 V.2.5) panels at the time of the meeting (*BRCA1*, *BRCA2*, *PALB2*, *CHEK2*, *ATM*, *RAD51C*, *RAD51D*, *BRIP1*, *MLH1*, *MSH2*, *MSH6* and *PMS2*). *BARD1* was also discussed, as this has been proposed as an addition to the R208 panel. GPVs in each of these genes confer an increased lifetime risk of different cancer types but pertinently in breast, ovarian, colorectal, endometrial, prostate and pancreatic cancers (table 1).

## Methods

### Pre-meeting preparations and survey

The organising committee (HH, KE, RJ, ZK, KS, BM and JC) comprised seven health professionals representing clinical genetics and oncogenetics. A preliminary survey was developed by the organising committee and distributed to the 24 RCGS from all four nations within the UK (24 Regional Services; 17 England and Royal Marsden Cancer Genetics Unit, 4 Scotland, 1 Wales and 1 Northern Ireland). This approach has been successfully used for previous UKCGG guidelines.^11^,^13^ The survey questions aimed to assess current practice for recontact and follow-up of at-risk individuals in RCGS across the UK.

A number of proposed consensus statements were developed by the organising committee based on the premeeting survey results.

### Format of consensus meeting

The consensus meeting was held online on Friday, 29 November 2024. Invitations were sent to representatives at all RCGS in the UK. The meeting was conducted in accordance with the UKCGG standard operating procedure (SOP) for consensus meetings. The meeting began with a series of presentations from experts to provide context-specific information and provided a forum for questions and discussion from delegates. The topics covered included a premeeting survey and audit of current recontact practice results, an overview of lifetime cancer risk estimates and current risk management guidelines for GPV, an NICPR overview and patient-facing clinical dashboards. This was followed by a series of polls on the proposed consensus statements, which delegates voted on by using the Slido platform. Each poll was closed when ≥80% of voting delegates had submitted a response. Consensus was deemed to be reached when ≥80% of respondents agreed on the statement posed, unless discussion led to the proposal of a revision to the wording of the statement, after which the poll was repeated with the revised wording to generate the final decision, including the possibility of no consensus. At least 50% of the starting number of voting delegates had to be present for each vote for the meeting to be quorate.

### Post-meeting communication

After the meeting, a summary document which included the agreed best practice statements was circulated to all attendees via email for their review and comment, to ensure that the statements were an accurate representation of the consensus reached.

### Meeting material availability

The UKCGG consensus meeting SOP (V.2), premeeting survey, meeting agenda, presentation slides and meeting report are available at: www.ukcgg.org/information-education/ukcgg-consensus-meetings.

## Results

### Premeeting survey

There were respondents from 21 RCGS in England, Scotland and Wales to the premeeting survey (table 2). Local databases (including Excel spreadsheets or local bespoke databases) were maintained at eight RCGS for individuals with a GPV in *BRCA1/2*, six RCGS for individuals with a GPV in *PALB2*, 11 RCGS for individuals with a GPV in MMR genes and six RCGS for individuals with a GPV in an intermediate penetrance hereditary breast or ovarian cancer gene (eg, *CHEK2*, *ATM*, *RAD51C, RAD51D* and *BRIP1*). 11 RCGS had no allocated time in staff job plans for inherited cancer predisposition database maintenance, while an average of 18.8 hours was allocated per week at RCGS for staff with protected time for database maintenance (range 4 hours to 40 hours per week). Seven RCGS made no contact with individuals with a GPV in a high penetrance CSG after their results appointment and subsequent referrals had been made. Five RCGS had a systematic recall for those with a GPV in a high-penetrance CSG. While there was slight variability between these centres, the approach was broadly consistent, with recall consisting of written recontact at specific timepoints corresponding to an individual’s age and CSG. For example, recontact of *BRCA1* and *BRCA2* carriers before age 30, around age 40 and at age 50 to reiterate recommendations for breast surveillance, gynaecological management and testing in children, respectively. Only one centre reported recontact at specific time intervals of 2–5 years. Four centres reported recalls on an ad hoc basis, such as when there is a change in published clinical guidelines. For individuals with a GPV in an intermediate penetrance CSG, there was no contact after their results appointment at 12 RCGS, with only four RCGS having a systematic recall system for this cohort.

**Table 2:** Summary of responses to the pre-meeting survey

Question	Regional clinical genetics service responses (n=21)
Do you maintain databases for individuals with GPVs in the following CSGs?	
*BRCA1/BRCA2*	Yes=8 (38%)
*PALB2*	Yes=6 (29%)
Intermediate penetrance hereditary breast or ovarian cancer genes (*CHEK2*, *ATM*, *RAD51C, RAD51D* and *BRIP1*)	Yes=6 (29%)
Mismatch repair genes	Yes=11 (52%)
Do you recontact individuals with GPVs in the following CSGs following their initial results appointment and appropriate onward referrals?	
High penetrance CSGs *(eg, BRCA1, BRCA2* and *PALB2*)	Yes=14 (67%) (systematic recall at five centres)
Intermediate penetrance CSGs (eg, *CHEK2*, *ATM*, *RAD51C, RAD51D* and *BRIP1*)	Yes=9 (43%) (systematic recall at four centres)

CSGs, cancer susceptibility genes; GPVs, germline pathogenic variants.

### Consensus meeting composition and working assumptions

The 53 voting delegates at the meeting comprised consultant clinical geneticists (n=21), genetic counsellors (n=21), patient representatives (n=4), NHS England representatives (n=4), specialty registrar in clinical genetics (n=1), a clinical scientist (n=1) and a research administrator (n=1). At least one representative was present from each of the 24 RCGS across all four UK nations. Six members of the organising committee were present at the meeting but did not vote.

Delegates were asked to vote for the best practice consensus statement, assuming:

- Appropriate resources are made available to deliver the clinical service or pathway proposed.
- That gene-specific statements are based on standard actionability variants for a given gene, that is, reduced or higher penetrance variants would be managed accordingly. For example, the *BRCA1* guideline statements presented would not be appropriate for an individual with a heterozygous *BRCA1* c.5096G>A (p.Arg1699Gln), a variant with recognised reduced penetrance.^14^ Variants with known reduced or increased penetrance need to be managed on a case-by-case basis with guidance provided by a specialist cancer genetics multidisciplinary team meeting.
- The recommended surveillance is based on current UKCGG guidelines, including an anticipated change in guidance, implemented in April 2025, recommending that all individuals with a GPV in *BRCA1/BRCA2/PALB2* are eligible for enhanced breast cancer surveillance from the age of 25 years.^15^ This is compared with the guidance in place at the time of the meeting, which stipulated that individuals with a GPV in *BRCA1/BRCA2/PALB2* were only eligible for breast cancer surveillance at age 25–30 years if they reached a 10-year risk of 8% or greater.

### Individuals with a GPV in *BRCA1*, *BRCA2* or *PALB2*

Consensus statements were discussed and voted upon by delegates for high-penetrance CSGs, *BRCA1*, *BRCA2* and *PALB2*. These are summarised below and are further detailed in online supplemental table 2.

#### Recontact

For all individuals with a GPV in *BRCA1*, *BRCA2* or *PALB2,* recontact should occur at the age of 25–30 years (where diagnosis was made at an earlier age) to reinforce information given at diagnosis in the form of patient-facing information and support.

#### Breast

For females ≥18 years with a GPV in *BRCA1*, *BRCA2* or *PALB2*, a referral to the very high-risk breast cancer screening (VHRS) programme or equivalent should be made at the time of diagnosis. For nations in which a VHRS register and recall system is not available, recontact should occur prior to the age at which surveillance is due to commence to arrange referral for appropriate breast surveillance.

#### Ovary

Further recontact regarding risk-reducing bilateral salpingo-oophorectomy (RRBSO) should occur at different ages depending on gene-specific ovarian cancer risk: 35–40 years (*BRCA1*) and 40–45 years (*BRCA2*). For females with a GPV in *PALB2*, RRBSO is only recommended where there is a ≥5% or lifetime risk of ovarian cancer based on family history. Consensus was reached that it is best practice to make recommendations for ovarian cancer risk management at diagnosis (if ≥18 years) and provide advice about seeking a reassessment if there are further ovarian cancer diagnoses in the family. Consensus was also reached for a further recontact at 50 years of age for individuals with a GPV in *PALB2* to remind them of the clinical management that should have occurred, ask if there are any new diagnoses of ovarian cancer in the family and offer advice for cascade testing in offspring.

#### Prostate/pancreas

Consensus was reached for recontact for males with a GPV in *BRCA2* at 40 years of age to provide advice on prostate screening. It was agreed that pancreatic screening advice should be given at the time of diagnosis for individuals with a GPV in *BRCA2*.

#### Final recontact

There was discussion regarding whether a final recontact should occur at 50 years of age for individuals with a GPV in *BRCA1* or *BRCA2* to ensure appropriate interventions had been discussed and/or implemented and to remind individuals about cascade testing for close relatives, but no consensus was reached.

### Individuals with a GPV in *ATM*, *CHEK2*, *RAD51C, RAD51D*, *BARD1* or *BRIP1*

Consensus statements were discussed and voted upon by delegates for intermediate penetrance CSGs (*ATM, CHEK2, RAD51C, RAD51D, BARD1* and *BRIP1*). These are summarised below and are further detailed in online supplemental table 3.

#### Breast

There was no consensus as to whether individuals with a GPV in an intermediate penetrance CSG (*ATM*, *CHEK2*, *RAD51C, RAD51D*, *BARD1* or *BRIP1*) should be proactively recontacted regarding breast cancer surveillance at any stage, assuming that appropriate advice and onward referrals had been made after their results appointment.

#### Ovary

Consensus was reached for recontact of females with a GPV in *RAD51C, RAD51D* and *BRIP1* at 50 years of age to provide information regarding RRBSO and to discuss their personal preferences.

#### Prostate

There was consensus that prostate cancer screening advice for males with a GPV in *ATM* or *CHEK2* should be given at the time of diagnosis, including advice to seek a reassessment if there were any further cancer diagnoses in the family or if they have specific questions about their genetic diagnosis.

#### Pancreas

It was agreed that pancreatic cancer screening advice should be given at the time of diagnosis for individuals with a GPV in *ATM*.

### Individuals with a GPV in *MLH1, MSH2, MSH6* or *PMS2*

The consensus meeting focused on long-term follow-up and recontact regarding gynaecological cancer risk management for individuals with a GPV in the MMR gene. Bowel cancer screening recontact and follow-up is arranged through the national NHS bowel screening programme in England via the National Lynch Register^16^ and was not discussed at this meeting. These recommendations are summarised below and are further detailed in online supplemental table 4.

#### Ovary and endometrium

Consensus was reached that recontact regarding RRBSO and total abdominal hysterectomy (TAH) should occur at 35–40 years of age in individuals with a GPV in *MLH1*, *MSH2* and *MSH6*, and regarding TAH only at 40–50 years of age for individuals with a GPV in *PMS2*.

#### Risk-reducing therapies

Consensus was reached that advice should be given regarding risk-reducing medication (aspirin) at the time of diagnosis.

#### Final recontact

There was discussion regarding whether a final recontact should occur at 50 years of age or similar for individuals with a GPV in an MMR gene to ensure appropriate interventions had been discussed and/or implemented and to remind individuals about cascade testing for close relatives, but no consensus was reached.

### Individuals with a GPV in a CSG identified in childhood (<18 years of age)

There was consensus that individuals found to have a GPV in *BRCA1, BRCA2, PALB2*, *MLH1, MSH2* or *MSH6* before the age of 18 years should be proactively offered an appointment with Clinical Genetics at 18 years of age (online supplemental table 5).

There was discussion but no consensus on whether an appointment with Clinical Genetics should be proactively offered to individuals when reaching adulthood if originally identified to have a GPV in *ATM*, *CHEK2*, *RAD51C, RAD51D, BARD1* or *BRIP1* during childhood.

### Content of recontact information

For all of the discussion, it was assumed that appropriate measures would be taken to check current patient details (address and whether the patient is alive/deceased) against central patient databases, such as the NHS spine, at the point of recontact.

It is recognised that individuals may have risk-reducing surgery at any time point following their genetic diagnosis and that surgery may take place in a different healthcare setting to the RCGS. Therefore, it was assumed that people who have had risk-reducing surgeries may not have been robustly removed from any recontact list, and as such, appropriate wording will need to be used in any communication with at-risk individuals. There was consensus that written communication, for example, by letter or email, would be the preferred method for recontact. It was agreed that it would be best practice to include information on risk-reducing surgery, cancer surveillance, risk-reducing therapies, reproductive options, family testing and research participation at each point of recontact. It was also agreed that it is best practice to signpost at-risk individuals at every recontact to available gene/condition-specific patient-facing resources to support their understanding of their condition and to facilitate engagement in their management. There was consensus acknowledgement that the information provided may vary depending on patient characteristics such as sex assigned at birth.

It was discussed that there may potentially be other times it would be best practice to recontact individuals, such as a clinical management guideline change or to flag research studies they may be eligible to take part in.

### Guidance on referral to other specialties

When referring individuals for risk-reducing mastectomy, risk-reducing gynaecological surgery or breast cancer surveillance, it was agreed that it is best practice to save the referral to the patient’s notes/record. There was no consensus reached as to whether it is necessary to ensure a referral for risk-reducing surgery or for breast cancer surveillance services had been received. There was consensus that it is not necessary to ensure risk-reducing surgery takes place following referral.

### Mechanism for recontact

There was no clear consensus on the preferred mechanism for recontact and follow-up, but there was broad agreement that input from both the national register(s) and local RCGS would be required. The majority of delegates felt that they would require additional clinical and administrative resources in order to implement these recommendations.

## Discussion

The premeeting survey highlighted significant variation in practices across RCGS regarding data collection, follow-up and recontact for individuals with GPVs in CSGs across the UK. Some RCGS allocated dedicated time for maintenance of clinical records and databases; others had no formalised processes for follow-up or recontact, particularly for individuals with a GPV in an intermediate penetrance CSG. The joint UKCGG/CanGene-CanVar consensus meeting involving experts representing all devolved nations and patient representatives aimed to standardise best practices, emphasising the need for proactive recontact in specific circumstances and structured follow-up pathways to ensure equitable care delivery across all regions and to ensure the opportunities to prevent and detect cancer earlier in this increased-risk group are optimised.

It was widely recognised by the consensus group that comprehensive counselling of specific cancer risks, symptom awareness and signposting to information is required at the results appointment. It was agreed that referrals for surveillance and risk-reducing surgeries should also be made at the time of results, if age appropriate. However, it was noted that due to the differing time points at diagnosis of an inherited cancer predisposition and ages of relevant interventions, best practice guidelines for additional recontact are required. Through structured discussion and polling among key UK stakeholders, we provide consensus best practice guidelines for recontact of these individuals (table 3).

**Table 3:** A summary of best practice guidelines for recontact of individuals with a heterozygous GPV in a breast and/or ovarian CSG

Best practice guidance regarding recontact	Key purpose of recontact*	Age of recontact
**High penetrance CSGs *****(BRCA1, BRCA2*** **or *****PALB2*****)**		
Recontact regarding breast cancer risk management	Reinforce information given at diagnosis in the form of patient-facing information and support. Ensure breast cancer risk-reducing interventions have been discussed/commenced.	25 years
Recontact regarding ovarian cancer risk management	Regarding RRBSO.	35–40 years (*BRCA1*) 40–45 years (*BRCA2*) and 50 years (PALB2)†
Recontact regarding prostate cancer risk management	Provide advice on prostate screening.	40 years (*BRCA2* only)
**Intermediate penetrance CSGs** ***(ATM, CHEK2, RAD51C, RAD51D, BARD1*** **and *****BRIP1*****)**		
Recontact regarding ovarian cancer risk management	Regarding RRBSO.	50 years (*RAD51C/D* and *BRIP1* only)
Mismatch repair genes (*MLH1*, *MSH2*, *MSH6* and *PMS2*)		
Recontact regarding risk-reducing gynaecological surgery	Regarding RRBSO and/or TAH.	35–40 years regarding RRBSO and TAH (*MLH1*, *MSH2* and *MSH6*) 40–50 years regarding TAH only (*PMS2*)

* At all points of recontact, the opportunity to reinforce information already given, provide key updates, and signpost to appropriate resources should be used.

† RRBSO only recommended where ≥5% lifetime risk of ovarian cancer.

CSGs, cancer susceptibility genes; GPVs, germline pathogenic variants; RRBSO, risk-reducing bilateral salpingo-oophorectomy; TAH, total abdominal hysterectomy.

Key consensus points established specific age-based recontact practices for high penetrance CSGs, *BRCA1, BRCA2* and *PALB2*, including recontact at 25 years to review breast cancer risk management and ovarian cancer risk management at gene-specific ages. However, no consensus was reached for recontact regarding breast cancer risk management in intermediate penetrance CSGs (*ATM*, *CHEK2*, *RAD51C, RAD51D* or *BARD1*). This likely reflects the heterogeneous group this represents, with significant influence from family history and non-genetic risk factors, with the need for individualised surveillance recommendations,^17^,^19^ such that formulating generalisable recommendations for recontact is challenging. The management of these individuals may also be modified by the introduction of polygenic risk scores and/or more routine assessment of breast density in the future.^13^

While consensus was not reached for recontact of individuals with a GPV in intermediate penetrance CSGs (*RAD51C* and *RAD51D*) with respect to breast cancer risk management, consensus was reached for recontact for these genes (and *BRIP1*) with respect to ovarian cancer risk management. The consensus for recontact of patients to reinforce information on RRBSO likely reflects the recognition by the group that early detection of ovarian cancer is significantly limited by non-specific symptoms and lack of proven surveillance, such that there was strong agreement to maximise the opportunity to reduce the risk of ovarian cancer occurrence.

For individuals with a GPV in *CHEK2* or *ATM*, we provide best practice guidance which favours providing information regarding prostate cancer risk at a results appointment with patient-initiated follow-up (PIFU) if and when required. The use of PIFU was also recommended for individuals with a GPV in *ATM* regarding pancreatic cancer risk management. Given ongoing studies in this area and the likelihood of additional surveillance methods and recommendations in the future, views on proactive recontact may change in the future.

Given the implementation of the national Lynch syndrome registry in England and direct referral of individuals with Lynch syndrome into a well-established national bowel cancer screening programme in England, the management of individuals with a GPV in the MMR gene was not extensively discussed. However, best practice guidance regarding gynaecological cancer risk management and delivery of advice regarding risk-reducing medication was reached. Future work may include the formation of consensus statements on the role of RCGS in the follow-up and recontact of individuals with hereditary bowel cancer risk, including those with Lynch syndrome.

Consensus was achieved for proactive recontact of individuals with a GPV in the MMR gene for gynaecological cancer risk management, but uncertainty remained around final recall practices. For childhood GPV findings, proactive recall for genetic counselling at age 18 years was recommended for high-penetrance CSGs, with no consensus for intermediate-penetrance CSGs.

The meeting emphasised the preference for written communication for recontact by meeting attendees. Future work to establish patient preference for recontact and preference through collaboration with patient groups would be of particular benefit. There was consensus that each recontact, regardless of its key aim, should be used as an opportunity to reinforce information already given regarding cancer surveillance, risk-reducing surgeries and therapies, cascade testing for family members and discussion of reproductive options. Furthermore, the development of new early cancer detection methods and the optimisation of existing technology are dependent upon research. The group therefore felt that recontact of individuals to invite them to research studies should be proactively encouraged.

The inclusion of CSGs in rapid foetal exome testing and genomic testing panels for childhood disorders will increase the number of incidental findings of GPV in CSGs with no clinical actionability during childhood in the heterozygous form.^20^ We provide best practice guidance on the recontact of these individuals. The implementation of this guidance presents challenges associated with follow-up, with a lag time of up to 18 years. Sufficiently resourced recall systems will be required to ensure robust and equitable recall for these high-risk individuals.

Even for those tested during adulthood, the robust recall of an individual with a GPV in, for example, *PALB2* at 25–30 years for breast cancer surveillance, then again at 50 years of age regarding ovarian cancer risk management, requires careful coordination with inbuilt failsafes. Co-creation of recall systems with patient groups and close collaboration between clinicians and patients will be required to ensure our recommendations are equitably delivered. Technologies such as smartphone applications and artificial intelligence-powered chatbots could be used to deliver information in a bespoke and accessible manner for all. In addition, the advantage of a robust national registry, such as the NICPR, coupled with RCGS specialising in family liaison and tailored patient care, should be capitalised upon with the core aim of maximising early cancer detection in those with a hereditary cancer predisposition.

In answering the consensus statements, delegates were asked to vote, assuming that appropriate resources are made available to deliver the agreed recommendations from the meeting. A common theme of the discussion was that current local resources in RCGS are not adequate to deliver these recommendations. Different approaches to delivering the agreed recontact were discussed, including local, national or hybrid models. With the development of the NICPR, it was hoped that this national register of all individuals with a monogenic cancer predisposition could be used to support the delivery of these recommendations.

As the number of individuals identified with an inherited cancer predisposition increases over time, the infrastructure, training and specialised staffing requirements will need to evolve to meet this demand. The group discussed a hybrid model of standardised recall using national infrastructure and tailored delivery of information using local knowledge of individuals and their families through RCGS. This model would provide clinical robustness and efficiency through a national recall system as well as personalised and compassionate care through local networks. If appropriate resources can be secured, implementing these recommendations will necessitate sufficient administrative and clinical support, the development of failsafe recall systems, and collaboration with patient groups to ensure comprehensive, equitable care for hereditary cancer risk management.

## Supplementary material

10.1136/jmg-2025-110979 online supplemental file 1
