# Supplementary material for: Recontact and follow-up for individuals with germline pathogenic variants in hereditary breast and ovarian cancer susceptibility genes: a UK Cancer Genetics Group consensus meeting
Source: J Med Genet. 2025 Oct 1;63(1):e110979. doi: 10.1136/jmg-2025-110979 (PMC12772612; doi:10.1136/jmg-2025-110979)
Supplement: online supplemental file 1 [file jmg-63-1-s001.pdf]

Recontact and follow-up for individuals with germline pathogenic variants in hereditary breast and ovarian cancer susceptibility genes: a UK Cancer Genetics Group consensus meeting

Supplementary material

Supplementary Table 1 – summary of current UK Cancer Genetics Group guidance for the management of individuals with a germline pathogenic variant in breast and ovarian cancer susceptibility genes

Cancer susceptibility gene	Breast cancer risk management	Gynaecological cancer risk management	Other cancer risk management
BRCA1	*Surveillance:* Annual breast MRI from 25 – 50 years and annual mammogram from 40 years. Breast density review age 50 and MRI continued if BI-RADS classification B-D. Routine recall stops age 71, but can self-refer for annual mammogram over age 70 years*<br><br>*Risk-reducing surgery:* Bilateral risk-reducing mastectomy should be considered<br><br>*Risk-reducing endocrine therapy:* Not recommended	*Risk-reducing surgery:* Risk-reducing bilateral salpingo-oophorectomy (RRBSO) should be discussed and can be offered when childbearing is complete and no earlier than age 35–40 years	*Prostate:* The pros and cons of PSA screening should be discussed<br><br>*Pancreatic:* consider surveillance through research study
BRCA2	*Surveillance and risk-reducing surgery:* Same as *BRCA1*<br><br>*Risk-reducing endocrine therapy:* The pros and cons should be discussed	*Risk-reducing surgery:* RRBSO should be discussed and can be offered when childbearing is complete and no earlier than age 40-45 years	*Prostate:* Annual PSA from 40 years with onward referral if PSA >3.0ng/ml<br><br>*Pancreatic:* consider surveillance through research study
PALB2	*Surveillance and risk-reducing surgery:* Same as *BRCA1*	*Risk-reducing surgery:* For *PALB2* heterozygotes with ≥5% lifetime risk of ovarian cancer, RRBSO should be considered at 50 years, or younger dependent on an individualised risk assessment	*Pancreatic:* consider surveillance through research study

CHEK2	*Surveillance: Should be based on individualised risk assessment using tools such as CanRisk and in accordance with NICE guidelines on familial breast cancer (CG164) and VHRS guidelines* (13)<br><br>*Risk-reducing surgery:* Bilateral risk-reducing mastectomy can be considered if lifetime risk ≥30%<br><br>*Risk-reducing endocrine therapy:* can be considered within the context of family history		*Prostate:* PSA screening can be considered annually from age 50 years, or earlier if there is a significant family history of prostate cancer and following discussion of the pros and cons
ATM	*Surveillance, risk-reducing surgery and risk-reducing endocrine therapy:* Same as *CHEK2*		*Prostate:* PSA screening can be considered annually from age 50 years, or earlier if there is a significant family history of prostate cancer and following discussion of the pros and cons<br><br>*Pancreatic:* consider surveillance through research study
BARD1**	*Surveillance, risk-reducing surgery and risk-reducing endocrine therapy:* No UK guidelines at present but management principles expected to be equivalent to *CHEK2* and *ATM*		
RAD51C/D	*Surveillance and risk-reducing surgery:* Same as *CHEK2*	*Risk-reducing surgery:* For *RAD51C/D* heterozygotes with ≥5% lifetime risk of ovarian cancer, RRBSO can be considered in those younger than 50 years following an individualised risk assessment	
BRIP1		*Risk-reducing surgery:* same as	

		RAD51C/D	
MLH1* and *MSH2		*Risk-reducing surgery:* RRBSO and total abdominal hysterectomy (TAH) should be discussed and offered when childbearing is complete and no earlier than age 35-40 years	*Gastrointestinal:* 2-yearly colonoscopy from age 25 years until 75 and one-off H. pylori testing and eradication<br><br>*Pancreatic:* consider surveillance through research study<br><br>*Risk-reducing therapies:* Discuss the pros and cons of aspirin risk-reducing medication from the age of 18 until 60 years<br><br>*Diet:* Provide information on the benefits of increasing dietary fibre and recommend a daily intake of 30-35g/day<br><br>*Prostate:* PSA screening can be considered from age 40 years
MSH6		*Risk-reducing surgery:* Same as *MLH1* and *MSH2*	*Gastrointestinal:* 2-yearly colonoscopy from age 35 years until 75 years and one-off H. pylori testing and eradication<br><br>*Pancreatic, risk-reducing therapies and diet:* Same as *MLH1* and *MSH2*<br><br>*Prostate:* No recommendations
PMS2		*Risk-reducing surgery:* The pros and cons of postmenopausal TAH alone should be discussed.	*Gastrointestinal, risk-reducing therapies and diet:* Same as *MSH6*<br><br>*Pancreatic and prostate:* No recommendations

Supplementary Table 1: Summary of UKCGG guidance*** on cancer risk management in individuals with a heterozygous GPV in the CSGs listed above (6).

*In England breast cancer surveillance for individuals with a GPV in *BRCA1*, *BRCA2* and *PALB2* or assessed as having an equivalent lifetime breast cancer risk are managed through the Very High Risk Screening (VHRS) programme (13)

***BARD1* is not routinely tested in the UK but will likely be added to the national genomic test directory in England in the near future with national guidelines expected to be equivalent to *ATM* or *CHEK2* breast cancer management recommendations.

***UKCGG summary guideline versions used as reference are: *BRCA1 version 2 (31/3/2023), BRCA2 version 2 (31/3/2023), PALB2 version 1 (31/03/2023), CHEK2 version 2 (4/2/2025), ATM version 2 (4/2/2025), RAD51C version 1 (13/12/2022), RAD51D version 1 (13/12/2022), BRIP1 version 1 (31/3/2023), Lynch syndrome genes Version 4 (Nov 2024)*

Supplementary Table 2 - high penetrance cancer susceptibility gene statement outcomes

Statement	Voting breakdown
BRCA1	
For individuals with a GPV in *BRCA1*, there are times when it would be best practice to proactively recontact the patient (specific scenarios to be discussed in next questions).<br>***44 responses***	**Consensus reached 98%**<br>Yes (43), No - all appropriate advice should be provided at diagnosis (0), Not sure (1)
For individuals with a GPV in *BRCA1*, it is best practice to refer for VHRS breast screening at diagnosis (if >18yrs), with patient consent.<br>***43 responses***	**Consensus reached 93%**<br>Yes (40), No (1), Not sure (2)
For individuals with a GPV in *BRCA1*, if VHRS referral is made at diagnosis, it is best practice to proactively recontact the patient when they are approaching 25 years of age to ensure that they are aware that they will be invited for VHRS and that they will need to make recontact if they do not receive an invitation.<br>***45 responses***	**Statement reworded (see next question)**<br>Yes (25), No - appropriate advice should be provided at diagnosis (12), Not sure (8)
It would be best practice to proactively recontact individuals with a GPV in *BRCA1* between the ages of 25-30 years to reinforce information given at diagnosis in the form of patient facing information and support.<br>***43 responses***	**Consensus reached 88%**<br>Yes (38), No (3), Not sure (2)
For individuals with a GPV in *BRCA1*, where a national VHRS register and recall system is not in place (e.g. Scotland, Wales), it would be best practice to recontact the patient to facilitate referral prior to the age at which surveillance would commence.<br>***43 responses***	**Consensus reached 93%**<br>Yes (40), No (0), Not sure (3)

For individuals with a GPV in *BRCA1*, it is best practice to proactively recontact the patient to remind them of recommendations for risk-reducing bilateral salpingo-oophorectomy (age to be discussed in next question).<br>***43 responses***	**Consensus reached 88%**<br>Yes (38), No - appropriate advice should be provided at diagnosis (2), Not sure (3)
For individuals with a GPV in *BRCA1*, it is best practice to proactively recontact the patient to remind them of recommendations for risk-reducing bilateral salpingo-oophorectomy by:<br>***45 responses***	**Consensus reached for 35-40 years**<br>35 years of age (10), 40 years of age (6), 37 years of age (27), Not sure (2)
For individuals with a GPV in *BRCA1*, it is best practice to proactively recontact the patient for a final time by 50 years of age to remind them of the clinical management that should have occurred and to offer advice for cascade testing.<br>***46 responses***	**No consensus reached**<br>Yes (19), Yes - but a different age (3), No (16), Not sure (8)
BRCA2	
For individuals with a GPV in *BRCA2*, we should undertake the same level and type of recontact as *BRCA1* with the exception of the age to contact about risk-reducing gynaecological intervention and potential additional recontact for prostate/pancreatic advice<br>***36 responses***	**Consensus reached 100%**<br>Yes (36), No (0), Not sure (0)
For individuals with a GPV in *BRCA2*, it is best practice to proactively recontact the patient to remind them of recommendations for risk-reducing bilateral salpingo-oophorectomy by:<br>***36 responses***	**Consensus for 40-45 years**<br>40 years of age (22), 45 years of age (15), 50 years of age (0), Not sure (0)
For individuals with a GPV in *BRCA2*, it is best practice to proactively recontact the patient when they are approaching 40 years of age to ensure that they are aware that prostate cancer screening may be available.<br>***36 responses***	**Consensus reached 94%**<br>Yes (34), Yes - but a different age (1), No - appropriate advice should be provided at diagnosis (0), Not sure (1)

For individuals with a GPV in *BRCA2*, it is best practice to discuss pancreatic surveillance recommendations at diagnosis & provide advice about re-assessment if further cancer diagnoses in the family/if they have specific questions about their genetic diagnosis<br>***35 responses***	**Consensus reached 86%**<br>Yes (30), No (2), Not sure (3)
PALB2	
For *PALB2*: Do you accept previous *BRCA1/2* answers for proactive recontact at 25-30 years of age?<br>***38 responses***	**Consensus reached 97%**<br>Yes (37), No (1), Not sure (0)
For individuals with a GPV in *PALB2*, it is best practice to make recommendations for ovarian cancer risk management at diagnosis (if >18yrs) and provide advice about seeking a re-assessment if there are further ovarian cancer diagnoses in the family?<br>***36 responses***	**Consensus reached 92%**<br>Yes (33), No (2), Not sure (1)
For individuals with a GPV in *PALB2*, it is best practice to proactively recontact the patient by 50 years of age to remind them of the clinical management that should have occurred; ask if there are any new diagnoses of ovarian cancer in the family; and to offer advice for cascade testing in children.<br>***40 responses***	**Consensus reached 83%**<br>Yes (33), Yes - but a different age (1), No - appropriate advice should be provided at diagnosis (3), Not sure (2)

Supplementary Table 3 - intermediate penetrance cancer susceptibility gene statement outcomes

Statement	Voting breakdown
ATM, CHEK2, RAD51C/D and BARD1	
For purposes of breast surveillance, we should have the same recontact recommendations for all individuals with a GPV in an intermediate penetrance breast CSG; *ATM*, *CHEK2*, *RAD51C/D* and *BARD1*?<br>***41 responses***	**Statement reworded (see next question)**<br>Yes (26), No (13), Not sure (2)
It is best practice to offer proactive recontact to individuals with a GPV in an intermediate penetrance breast CSG (*ATM*, *CHEK2*, *RAD51C/D*, *BARD1*) relating to breast cancer surveillance and to provide additional information.<br>***40 responses***	**No consensus reached**<br>Yes (10), No - information should be provided at diagnosis (25), Not sure (5)
ATM	
For individuals with a GPV in *ATM*, it is best practice to discuss prostate surveillance recommendations at diagnosis and provide advice about seeking a re-assessment if there are further cancer diagnoses in the family or if they have specific questions about their genetic diagnosis.<br>***38 responses***	**Consensus reached 97%**<br>Yes (37), No (1), Not sure (0)
For individuals with a GPV in *ATM*, it is best practice to discuss pancreatic surveillance recommendations at diagnosis and provide advice about seeking a re-assessment if there are further cancer diagnoses in the family or if they have specific questions about their genetic diagnosis.<br>***36 responses***	**Consensus reached 94%**<br>Yes (34), No (0), Not sure (2)
CHEK2	

For individuals with a GPV in *CHEK2*, it is best practice to discuss prostate surveillance recommendations at diagnosis and provide advice about seeking a re-assessment if there are further cancer diagnoses in the family or if they have specific questions about their genetic diagnosis.<br>***36 responses***	**Consensus reached 97%**<br>Yes (35), No (0), Not sure (1)
RAD51C/D and BRIP1	
For individuals with a GPV in *RAD51C*, it is best practice to proactively recontact the patient to remind them of recommendations for risk-reducing bilateral salpingo-oophorectomy (age to be discussed in next question).<br>***38 responses***	**Consensus reached 87%**<br>Yes (33), No (4), Not sure (1)
For individuals with a GPV in *RAD51C*, it is best practice to proactively recontact the patient by 50 years of age to remind them of the option of risk-reducing bilateral salpingo-oophorectomy and to reinforce previous advice.<br>***48 responses***	**Consensus reached 84%**<br>Yes (32), Yes - but a different age (1), No - appropriate advice should be provided at diagnosis (5), Not sure (0)
For individuals with a GPV in *RAD51D* or *BRIP1* genes, do you accept previous *RAD51C* answers to undertake recontact and the age of recontact?<br>***38 responses***	**Consensus reached 97%**<br>Yes (37), No (1), Not sure (0)
MMR genes (MLH1, MSH2, MSH6 or PMS2)	
For individuals with a GPV in a MMR gene, it is best practice to make recommendations for risk-reducing medication (if >18yrs) at the time of diagnosis.<br>***34 responses***	**Consensus reached 100%**<br>Yes (34), No (0), Not sure (0)
For individuals with a GPV in *MLH1*, *MSH2*, *MSH6* (BSO/TAH) and *PMS2* (TAH only) it is best practice to proactively recontact the patient to remind them of the option of risk-reducing gynae surgery (age to be discussed in next question).	**Consensus reached 94%**<br>Yes (33), No (2), Not sure (0)

35 responses	
For individuals with a GPV in *MLH1*, *MSH2* or *MSH6*, it is best practice to proactively recontact the patient to remind them of option of risk-reducing gynae surgery by:<br>***36 responses***	**Consensus for 35-40 years**<br>35 years of age (11), 40 years of age (24), 45 years of age (1), Other (0)
For individuals with a GPV in *PMS2,* it is best practice to proactively recontact the patient to remind them of option of risk-reducing gynae surgery (TAH) by:<br>***37 responses***	**Consensus for 45-50 years**<br>35 years of age (1), 40 years of age (5), 45 years of age (11), 50 years of age (18), Other (2)
For individuals with a GPV in a MMR gene, it is best practice to proactively recontact the patient for a final time by 50 years of age to reinforce information given at diagnosis in the form of patient facing information and support<br>***38 responses***	**No consensus reached**<br>Yes (11), Yes - but a different age (0), No - appropriate advice should be provided at diagnosis (23), Not sure (4)

Supplementary Table 4 - mismatch repair gene statement outcomes

Statement	Voting breakdown
MMR genes (MLH1, MSH2, MSH6 or PMS2)	
For individuals with a GPV in a MMR gene, it is best practice to make recommendations for risk-reducing medication (if >18yrs) at the time of diagnosis.<br>***34 responses***	**Consensus reached 100%**<br>Yes (34), No (0), Not sure (0)
For individuals with a GPV in *MLH1*, *MSH2*, *MSH6* (BSO/TAH) and *PMS2* (TAH only) it is best practice to proactively recontact the patient to remind them of the option of risk-reducing gynae surgery (age to be discussed in next question).<br>***35 responses***	**Consensus reached 94%**<br>Yes (33), No (2), Not sure (0)
For individuals with a GPV in *MLH1*, *MSH2* or *MSH6*, it is best practice to proactively recontact the patient to remind them of option of risk-reducing gynae surgery by:<br>***36 responses***	**Consensus for 35-40 years**<br>35 years of age (11), 40 years of age (24), 45 years of age (1), Other (0)
For individuals with a GPV in *PMS2,* it is best practice to proactively recontact the patient to remind them of option of risk-reducing gynae surgery (TAH) by:<br>***37 responses***	**Consensus for 45-50 years**<br>35 years of age (1), 40 years of age (5), 45 years of age (11), 50 years of age (18), Other (2)
For individuals with a GPV in a MMR gene, it is best practice to proactively recontact the patient for a final time by 50 years of age to reinforce information given at diagnosis in the form of patient facing information and support<br>***38 responses***	**No consensus reached**<br>Yes (11), Yes - but a different age (0), No - appropriate advice should be provided at diagnosis (23), Not sure (4)

Supplementary Table 5 - general statements on recontact and referral pathway guidance outcomes

Statement	Voting breakdown
Recontact	
When recontacting individuals with a GPV in a CSG, the information provided may vary according to consideration of patient characteristics (e.g. sex/gender)<br>***35 responses***	**Consensus reached 89%**<br>Yes (31), No (1), Not sure (3)
When recontacting a patient, please rank the following by preference (1 being most preferred option to 3 being least preferred option).<br>***42 responses***	**1. Written communication: e.g. letter or email 2.95***<br>2. Other 1.26<br>3. Phone call 0.98
There would potentially be other times it would be best practice to recontact the patient (in addition to the scenarios discussed today), such as if there is a guideline change or to flag research studies they may be eligible to take part in.<br>***37 responses***	**Consensus reached 95%**<br>Yes (35), No (1), Not sure (1)
When recontacting a patient, it is best practice to include a reminder that clinical genetics can be recontacted for advice on (in accordance with relevant UKCGG recommendations):<br>***43 responses***	**Consensus reached for all below**<br>Family testing - 43 votes, 100%<br>Patient facing resources and information - 43 votes, 100%<br>Cancer risk-reducing surgery - 42 votes, 98%<br>Surveillance - 42 votes, 98%<br>Reproductive options - 42 votes, 98%<br>Risk-reducing medication - 38 votes, 88%<br>Research participation - 38 votes, 88%

At diagnosis, and when undertaking recontact, it is best practice to signpost individuals with a GPV in a CSG to gene/condition specific patient facing resources to support their understanding of their condition and to facilitate engagement in their management.<br>***33 responses***	**Consensus reached 100%**<br>Yes (33), No (0), Not sure (0)
Referrals to other specialties	
When referring patients to the Very High Risk Breast Screening programme (or equivalent), it is best practice to save confirmation of the referral to the patients notes/record.<br>***39 responses***	**Consensus reached 97%**<br>Yes (38), No (0), Not sure (1)
When referring patients to the Very High Risk Breast Screening programme (or equivalent), it is best practice to check referral has been received and save referral receipt confirmation to the patients notes/record.<br>***37 responses***	**No consensus reached**<br>Yes (15), No (16), Not sure (6)
When referring patients for risk-reducing mastectomy, it is best practice to save the confirmation of the referral to the patients notes/record.<br>***40 responses***	**Consensus reached 98%**<br>Yes (39), No (0), Not sure (1)
When referring patients for risk-reducing mastectomy, it is best practice to confirm receipt of the referral and save the referral receipt confirmation to the patient's notes/record.<br>**41 responses**	**No consensus reached**<br>Yes (10), No (27), Not sure (4)
When referring patients for risk-reducing mastectomy, it is best practice to confirm the surgery has been undertaken<br>***37 responses***	**Consensus reached 81%**<br>Yes (2), No (30), Not sure (5)
When referring patients for risk-reducing gynaecological surgery, it is best practice to save the referral to the patients notes/record<br>***36 responses***	**Consensus reached 100%**<br>Yes (36), No (0), Not sure (0)
When referring patients for risk-reducing gynaecological surgery, it is best practice to confirm receipt of the referral	**No consensus reached**<br>Yes (8), No (27), Not sure (1)

and save referral receipt confirmation to the patients notes/record<br>***36 responses***	
When referring patients for risk-reducing gynaecological surgery, it is best practice to confirm the surgery has been undertaken.<br>***33 responses***	**Consensus reached 85%**<br>Yes (4), No (28), Not sure (1)
Pathways and resources	
When a LP/P variant has been identified in childhood for which of the genes discussed today is it best practice to proactively offer an appointment age 18 years to discuss surveillance, risk-reducing interventions and reproductive options?<br>***30 responses***	**Consensus reached for:**<br>*BRCA1* - 30 votes, 100%<br>*BRCA2* - 30 votes, 100%<br>*PALB2* - 30 votes, 100%<br>*MLH1* - 30 votes, 100%<br>*MSH2* - 30 votes, 100%<br>*MSH6* - 29 votes, 97%<br>**No consensus reached for:**<br>*ATM* - 22 votes, 73%<br>*CHEK2* - 22 votes, 73%<br>*RAD51C* - 22 votes, 73%<br>*RAD51D* - 22 votes, 73%<br>*BRIP1* - 22 votes, 73%
If there is appropriate resource, infrastructure and clinical governance in place to support the pathway, please rank the following by preference (1 being most preferred option to 3 being least preferred option)<br>***44 responses***	**1. Recontact undertaken solely through the national register (with advice for patient to contact local centre for questions and follow-up) 3.5***<br>**2. Recontact undertaken by a combination of national register and local services 2.41**<br>3. Recontact undertaken solely by local regional genetics service 1.98<br>4. Other 0.59
My centre would require additional clinical and administrative resource to implement these recommendations<br>***39 responses***	**Consensus reached 95%**<br>Yes (37), No (0), Not sure (2)

*Each voting delegate ranked their preference for the statement. The highest ranked statement was given the highest score based on the number of options available. For 3 options, the first rank would receive 3 points, second rank would receive 2 points, and third rank would receive 1 point. The mean of the points is then calculated for each statement across all voting delegates.
